# Pneumonia and Pleural Empyema due to a Mixed *Lactobacillus* spp. Infection as a Possible Early Esophageal Carcinoma Signature

**DOI:** 10.3389/fmed.2016.00042

**Published:** 2016-09-28

**Authors:** Eleftheria Chaini, Nikolaos D. Chainis, Anastasios Ioannidis, Maria Magana, Chryssoula Nikolaou, Joseph Papaparaskevas, Melina-Vassiliki Liakata, Panagiotis Katopodis, Leonidas Papastavrou, George P. Tegos, Stylianos Chatzipanagiotou

**Affiliations:** ^1^Pulmonary Department, Corfu General Hospital, Corfu, Greece; ^2^Pulmonary Department, Athens Medical Center – Peristeri, Peristeri, Greece; ^3^Department of Nursing, Faculty of Human Movement and Quality of Life Sciences, University of Peloponnese, Sparta, Greece; ^4^Department of Biopathology and Clinical Microbiology, Aeginition Hospital, Medical School, National and Kapodistrian University of Athens, Athens, Greece; ^5^Department of Microbiology, Medical School, National and Kapodistrian University of Athens, Athens, Greece; ^6^Department of Biopathology and Clinical Microbiology, Athens Medical Center – Peristeri, Peristeri, Greece; ^7^Department of Pathology, Athens Medical Center – Peristeri, Peristeri, Greece; ^8^Department of Cardiac Surgery, Athens Medical Center, Athens, Greece; ^9^Torrey Pines Institute for Molecular Studies, Port St. Lucie, FL, USA; ^10^Wellman Center for Photomedicine, Massachusetts General Hospital, Boston, MA, USA; ^11^Department of Dermatology, Harvard Medical School, Boston, MA, USA

**Keywords:** *Lactobacillus delbrueckii*, *Lactobacillus gasseri*, pneumonia, pleural empyema, bacterial translocation, esophageal carcinoma

## Abstract

Lactobacilli are human commensals found in the gastrointestinal and genitourinary tract. Although generally conceived as non-pathogenic microorganisms, the existence of several reports implicating them in certain severe pathological entities renders this species as opportunistic pathogens. The case of a 58-year-old woman with mixed *Lactobacillus* infection is described. The patient was admitted in an outpatient clinic with community acquired pneumonia, and on the third day of hospitalization she presented rapid pneumonia deterioration. Subsequent imaging techniques revealed increased pleural empyema in alignment with the general deterioration of her clinical condition. Pleural fluid culture revealed the presence of *Lactobacillus delbrueckii* and *Lactobacillus gasseri* and the infection was successfully treated with clindamycin. Five months after hospital discharge and an overall good condition, the patient developed signs of dysphagia and upon re-admission an inoperable esophageal carcinoma was diagnosed. The patient succumbed to the cancer 11 months later. Herein, we report for the first time a mixed respiratory infection due to lactobacilli, possibly associated with a formerly unveiled esophageal malignancy.

## Introduction

Herein, we report the case of a woman who developed pneumonia and empyema due to a mixed *Lactobacillus delbrueckii* and *Lactobacillus gasseri* infection. Intriguingly, this mixed lactobacilli lung and pleural infection was associated with a formerly unveiled esophageal malignancy.

### Case Report

A 58-year-old woman (smoker, 30 packs/year) with no past medical history was admitted to the outpatient Pulmonary Department of the Athens Medical Center for the evaluation of fever and dyspnea 4 days before admission. Two days before, the patient received empirical treatment with orally administered moxifloxacin (400 mg/day) and oseltamivir (75 mg/12 h) for community acquired pneumonia (CAP). Upon admission, clinical and laboratory testing as well as echocardiogram, chest X-ray (CXR), and chest computerized tomography (CCT) with intravenous contrast material were performed.

**Figure 1:**
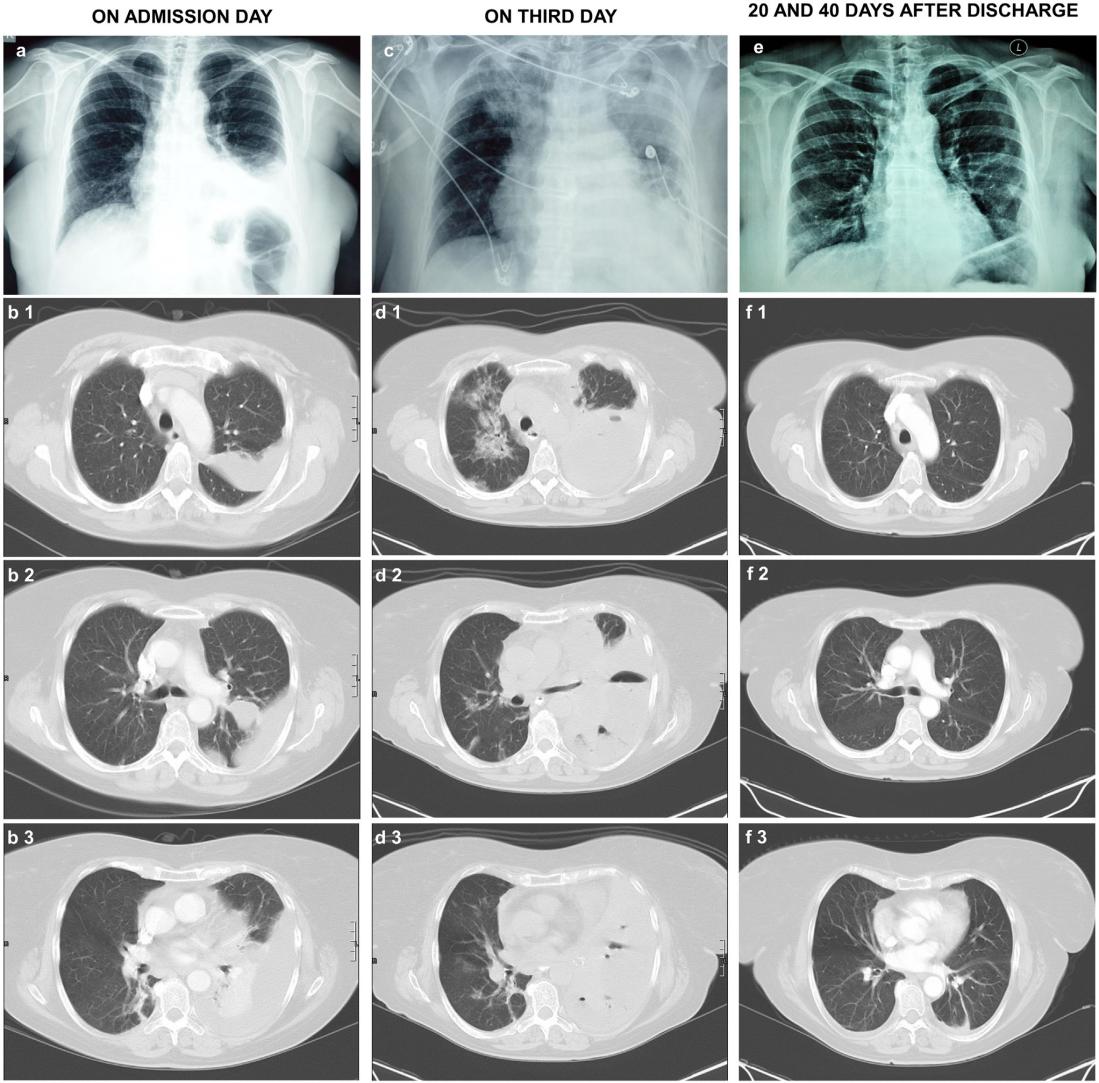
**(A)** CXR and **(B1–B3)** CCTs with intravenous contrast material on the admission day exhibit left-sided pleural fluid with consolidation and atelectasis of the left lower lung field. **(C)** CXR and **(D1–D3)** CCTs on the third day of admission. They exhibit significant increase of the left-sided pleural effusion with compressive atelectasis of the left lung and development of extensive consolidations in the right lung, especially in the upper lung fields. Small air collections are present in the pleural fluid. The chest drainage tube can be seen on the left of the CXR. **(E)** CXR 20 days and **(F1–F3)** CCT 40 days after the patient discharge from the hospital. They show complete resolution of the lung consolidations, limited left pleural thickening, and minimal pleural fluid (**1**: upper lung fields, **2**: middle lung fields, **3**: lower lung fields). CXR, chest X-ray; CCT, chest computerized tomography.

On examination upon first admission, the patient appeared with normal appetite and no apparent weight loss. She was febrile (38.5°C) without chills and hypoxic with oxygen saturation (SO_2_) of 94% on room air. On auscultation, dullness and decreased breath sounds were noted over the left lower chest. The echocardiogram was normal. Her blood tests demonstrated elevated white blood cells (WBC) count (10,760 cells/μL, neutrophils 81%), erythrocyte sedimentation rate (ESR) (102 mm/h, control < 30 mm/h), and C-reactive protein (CRP) (36.1 mg/dL, control < 0.5 mg/dL). Repeated blood cultures and urinary antigen tests for *Pneumococcus* and *Legionella* were negative. CXR (Figure 1A) and CCT (Figure 1B) on the admission day showed left-sided pleural effusion with consolidation and atelectasis of the left lower lung field.

The patient continued the treatment for CAP complicated by pleural effusion, with intravenous moxifloxacin (400 mg/day) and supplemental oxygen therapy. However, on the third day of hospitalization, her clinical condition dramatically deteriorated with severe dyspnea, without orthopnea (respiratory rate 30 breaths/min), hypoxemia (SO_2_ of 92% on a FiO_2_ 100%), and fever (38.9°C). The new CXR (Figure 1C) and CCT (Figure 1D) revealed a significant increase of the left-sided pleural effusion with compressive atelectasis of the left lung and development of extensive consolidations in the right lung, especially in the upper lung fields. Moxifloxacin was discontinued and followed by the administration of intravenous meropenem (1 g/8 h), amikacin (500 mg/12 h), and linezolid (500 mg/12 h).

Chest drainage was performed and pleural fluid analysis exhibited elevated WBC count (14,850 cells/μL, neutrophils 95%) and lactate dehydrogenase (LDH) levels (1257 U/L), decreased levels of glucose (2 mg%), and pH 7.0. Pleural fluid Gram-stain showed the presence of Gram-positive bacilli and was both aerobically and anaerobically cultured. After 48 h incubation, two morphologically distinct types of Gram-positive rods colonies appeared. The bacterial isolates were oxidase-negative, catalase-negative, and non-motile. Both isolates were preliminary identified as *Lactobacillus* spp.

Species identification was performed using a previously described 16S polymerase chain reaction (PCR) and sequencing protocol (1, 2). Total DNA was extracted from bacterial colonies harvested from the agar plates using the commercially available QIAamp DNA Mini Kit (Qiagen, Hilden, Germany) and the tissue protocol, according to the manufacturer’s instructions. PCR was performed using the GoTaq Hot Start Colorless Master Mix (Promega Corp., Madison, WI, USA) and a MyCycler Thermal Cycler (Bio-Rad Laboratories MEPE, Athens, Greece) in 50 μL final reaction volumes. PCR products were visualized under UV illumination and purified using the NucleoSpin Gel and PCR Clean-up kit (Macherey Nagel GmbH, Düren, Germany). Sanger sequencing was performed in a 3130 Genetic Analyzer (Applied Biosystems Life Technologies Ltd., Paisley, UK). Sequencing results were aligned and edited using the Data Analysis in Molecular Biology and Evolution (DAMBE) software ver. 5.1.5 (University of Ottawa, Ottawa, ON, Canada). The consensus sequence results that were obtained were compared with those submitted to the GenBank using the BLAST algorithm. The isolates were identified as *L. delbrueckii* (16S accession number KU207148) and *L. gasseri* (16S accession number KU207149). The pure isolation of the *L. delbrueckii* and *L. gasseri* strains in the culture of pleural fluid and their further molecular identification were suggestive of extraintestinal colonization and infection by these two species. It was suspected that the two lactobacilli strains were involved in the clinical manifestations of pneumonia and empyema.

Susceptibility testing was performed according to the guidelines of the European Committee on Antimicrobial Susceptibility Testing (EUCAST) for Gram-positive anaerobes using the *E*-test diffusion method, on Mueller–Hinton agar supplemented with 5% mechanically defibrinated horse blood and 20 mg/L β-Nicotinamide adenine dinucleotide (β-NAD) (MH-F) for 48 h at 37°C in anaerobic conditions. Antibiotics with available breakpoints were tested [minimum inhibitory concentration (MIC) values in milligrams per Liter; S = susceptible, R = resistant]: ampicillin (0.5; S), amoxicillin–clavulanic acid (0.25; S), ampicillin–sulbactam (0.5; S), ticarcillin–clavulanic acid (0.25; S), piperacillin–tazobactam (0.25; S), imipenem (0.25; S), meropenem (0.25; S), clindamycin (0.25; S), and vancomycin (>32; R). Fluoroquinolones were not included as lactobacilli bear intrinsic resistance to this antimicrobial class and respective breakpoints are not available.

Following identification of *Lactobacillus* species and susceptibility testing, intravenous clindamycin (600 mg/6 h) was added to the antibiotic regimen. The patient presented steady improvement with complete symptom resolution and clearance of the abnormal findings in the CCTs (Figures 1E,F).

During a 5-month follow-up, referring to the preceding respiratory tract infection, the patient exhibited no clinical signs and symptoms, as ascertained by standard clinical examination and routine laboratory tests with normal values (blood cell count, ESR and CRP, blood glucose, creatinine, and enzymes). However, on the fifth month after hospital discharge, she developed signs of dysphagia. Subsequent gastroscopy and biopsy revealed an inoperable esophageal carcinoma (invasive squamous cell carcinoma–SCC) localized in the lower thoracic esophagus proximal to the gastroesophageal junction. Thorough retrospective reconsideration of the imaging examinations (CXR, CCT, and CT of the upper and lower abdomen) performed on admission and during hospitalization did not reveal any signs of prior esophageal malignant process. The patient succumbed to the cancer 11 months later.

## Background

Lactobacilli are human commensals of the gastrointestinal and genitourinary tract. They are facultative anaerobic, non-sporing, non-motile, lactic acid producing Gram-positive rods. *Lactobacillus* species safety in the production of food supplements has been established worldwide for their common use as probiotics that exert pronounced beneficial activity (3). Nevertheless, probiotics as viable organisms may contribute to host infection under specific circumstances. Pathological entities related with lactobacilli as opportunistic pathogens include several life-threatening conditions such as bacteremia, sepsis, endocarditis, pneumonia, abscess formation, peritonitis, urinary tract infections, meningitis, and Fournier’s gangrene (1–10). Bacteremia and endocarditis are the most common *Lactobacillus*-associated infections, while pneumonia and empyema are rarer (9, 11–13).

Research and pilot studies concerning the effectiveness of probiotic therapy in the prevention of nosocomial pneumonia in critically ill patients (ventilator-associated pneumonia, VAP) has provided ambiguous results, either supporting clinical benefit or no benefit at all. The beneficiary effect lies in the fact that probiotic supplements enhance the protective activity of the gut microbiome with subsequent upregulation of immune defense, leading to an overall reduction of pathogens growth (14, 15). However, the use of these low virulent microorganisms as probiotics in immunocompromised patients has also raised safety concerns when applied to patients (a) with known comorbidities (history of heart defects, cardiac valves and prostheses, gastrointestinal injury), (b) after invasive procedures (hematological or organ transplantation), and (c) under chronic immunosuppressing medication (16). Extensive use of probiotics even as part of the therapeutic protocol for specific pathological conditions without enough information about the susceptibility and the translocation rate of each strain could result in late diagnosed infections (3).

In 1989, Querol et al. described two cases of pneumonia and empyema out of 28 previously reported *Lactobacillus* infection cases (13), while in 2014, Doern et al. identified 11 cases of *Lactobacillus* pneumonia (12). A current review of the literature revealed 15 cases of *Lactobacillus* pneumonia, while empyema was reported in 4 out of the 15 cases (8, 9, 11–13, 17–25). In the 15 previously described cases of *Lactobacillus-*attributed pneumonia, the documented *Lactobacillus* species were *L. casei* ssp. *rhamnosus* (18, 19), *L. rhamnosus* (12, 17), and *L. casei* (22, 24). *L. delbrueckii* and *L. gasseri* are rarely identified as infection causative agents (26). However, there have been scarce reports linking the two commensals with bacteremia and urinary tract infections (5, 6, 11, 27). Four previously reported pneumonia and empyema cases were associated with underlying clinical conditions (gastro-pleural fistula, tracheo-esophageal fistula created by an esophageal carcinoma, impaired local lung immune system due to emphysema and bacteremia) (8, 9, 13, 17). The remaining 11 cases of pneumonia were associated with chronic myeloid leukemia, heavy smoking and pulmonary emphysema, neutropenia, transmission of *Lactobacillus* by probiotics in patient with lung transplantation, acquired immune deficiency syndrome (AIDS), VAP in a critically ill trauma patient, and with probiotic-associated aspiration (12, 18–25).

Pulmonary empyema, and pyopneumopericardium, caused by commensals of the upper respiratory tract (*Pseudomonas aeruginosa, Streptococcus* group D and G) have been previously associated with esophageal cancer (4). The most common form of esophageal cancer is SCC, a malignant epithelial tumor with squamous cell differentiation (28). Smoking and alcohol consumption have been among the major risk factors, while the carcinogenicity of the human papillomavirus (HPV) has been also associated with the disease (29). Esophageal cancer is usually advanced at presentation, and therefore it accounts as the eighth cause of cancer worldwide (30). The most common symptom of a patient with SCC is dysphagia; however, the assessment of esophageal cancer is not always easy due to the increasing number of asymptomatic patients. Additionally, it may be underdetermined by typical diagnostic tools; barium esophagography is only proceeded in symptomatic patients but the confirmation of the diagnosis is made through esophagogastroscopy and biopsy (7). Overall, no specific data exist concerning the time elapsing between the onset of the malignancy and the clinical manifestations.

When it comes to the association of cancer with the use of *Lactobacillus* species as probiotics, literature supports the protective role of these supplements toward several types of cancer. The protective activity of *Lactobacillus* species have been profound in colorectal, urinary bladder, and breast cancer, mainly due to the cytotoxic and antiproliferative role of their fermentation products against cancer cells (31–33). However, *Lactobacillus*-associated infections have been implicated in the land of preexisting malignancy, constituting severe medical conditions. To date, only one case of esophageal carcinoma associated with *Lactobacillus* pneumonia has been reported (13).

Lactobacilli have been placed among the prevailing bacterial milieu of the upper and lower esophageal mucosal linings (34, 35). The rather uncommon localization of *Lactobacillus* isolates in the lung could be therefore attributed to passive translocation through a possible communication of the anatomical structures. Indeed, a previously reported case of *Lactobacillus*-associated pneumonia complicated by empyema was secondary to a defined tracheo-esophageal fistula created by esophageal carcinoma in a patient tagged with severe risk factors (smoking more than 40 packs/year, heavy alcohol consumption, septic mouth due to multiple carious teeth, and hypoproteinaemia). In both cases, the fatal clinical course was attributed to the basal status of the two patients and not in the *Lactobacillus* pathogenicity (13). It should be noted that in both patients primary imaging techniques were negative for abnormal findings suggestive of esophageal cancer. In our case, after the diagnosis of esophageal cancer, independent revaluation of the CCTs by two radiologists confirmed the absence of prior abnormalities. In addition, CT with contrast material of the upper and lower abdomen did not present findings suggestive of malignancy at an earlier stage.

## Informed Consent Statement

Written informed consent is not required if data is drawn from observed behavior or if data does not contain identifying information, as it is in the present report.

## Discussion

Lactobacilli are ubiquitous commensals of the mucosal surfaces of the gastrointestinal and genitourinary tract. Despite being rare, there has been reported causality between the bacteria and various infections in immunocompromised individuals. However, in most cases, the true prevalence of lactobacilli-associated infections is underestimated as the bacterium is typically regarded as commensal or occasional contamination; thus, debate exists concerning the clinical significance of lactobacilli isolation from normally sterile specimens.

While most of the pneumonia cases are attributed to single bacterial or viral infection, there have been reports on mixed CAP cases that follow the general pattern of (i) dual viral, (ii) viral plus bacterial, and (iii) dual bacterial. In the latter pattern, a *Streptococcus pneumoniae* infection is usually accompanied by *Haemophilus influenzae* or an atypical pathogen (*Legionella pneumoniae, Chlamydia pneumoniae*, and *Mycoplasma pneumoniae*) (36–38). In this case, the causal relationship of *L. delbrueckii* and *L. gasseri* isolates with pneumonia and empyema is supported for the first time, suggesting coinfection from different species of the same genus. The case of pneumonia and empyema, with the partition of both lactobacilli isolated from a sterile site along with the systemic inflammatory reaction confirmed by laboratory evaluation and a molecular technique, strongly suggests the two bacterial species as the causative microorganisms of the mixed infection. However, the etiology defining the pathogenetic background of the infection and the lactobacilli portal of entry is rather puzzling. The existence of a mixed *Lactobacillus* infection and the possible correlation with the pre-existence of a malignancy was the central observation in this case. Lab and clinical findings are influenced by two major risk factors: (a) heavy smoking directly related with the potential of preexisting undiagnosed esophageal carcinoma and (b) moxifloxacin treatment. Regarding the patient’s history, she denied any previous use of probiotic preparations, and the provided history did not reveal interventions that could alter the normal gastrointestinal flora. Therefore, the possibility of acquiring the infectious strains through prior use of probiotic preparations or from ingested foods may be considered insignificant.

The antibiotic treatment may also cause lactobacilli selection through an uncharted infection acquisition mechanism. Prior ineffective therapy with moxifloxacin could be considered as a risk factor for *Lactobacillus* population resistance, bacteremia, and translocation (39, 40). The susceptibility profile of *L. delbrueckii* and *L. gasseri* was similar with those previously reported. Lactobacilli are generally susceptible to penicillin, ampicillin, clindamycin, erythromycin, and cephalothin (5, 13). Antibiotics modify the intestinal flora thus enhancing the procedure of translocation; the presence of the mixed lactobacilli population could be attributed in this mechanism (41).

Empyema constitutes a life-threatening infection that is associated with several underlying medical conditions such as lung cancer, alcoholism, and conditions that carry the risk of aspiration (critically ill patients, comatose patients, neurological disorders). In the post-antibiotic era, literature is suggestive that high isolation rates of anaerobic Gram-positive bacteria from pleural specimens imply their leading role in most cases of empyema (42, 43). Anaerobic infections usually derive from the relatively low virulent normal flora that is prevalent in infections associated with predisposing or underlying medical conditions, thus giving the opportunistic pathogens the ability to establish infection through deeper tissue penetration. Another hint, which is supported by literature and is also present in this case, is the fact that opportunistic pathogens are often mixed with other anaerobic or aerobic bacteria (44). Therefore, the immunomodulatory impact of lactobacilli along with pleural fluid settlement through blood flow could be indirectly indicative of an underlying disease and the absence of overt esophageal carcinoma symptoms either before or during hospitalization does not exclude the possibility of underlying malignancy.

## Concluding Remarks

To date, this is the first report of a mixed CAP case complicated by empyema which is attributed to different species of the same genus. Additionally, *L. delbrueckii* and *L. gasseri* are herein implicated in the pathogenesis of a complicated lower respiratory tract infection. Despite the common belief that that lactobacilli are uncommon pathogens, the identification in extra-intestinal or extra-genitourinary sites of infection should not be overlooked. This case indicates that the detection of lactobacilli may serve as a probable early sign of an underlying but not yet unveiled potentially fatal condition.

The fatal clinical course in the majority of the cases complicated with *Lactobacillus*-associated infections is attributed to the underlying status. This case corroborates the aspect that microbial pathogenicity is indefinable and depends on the *ad hoc* host–microbe interaction. In clinical cases associated with infectious disorders, every microorganism isolated from naturally sterile clinical specimens, should be considered as a putative causative agent warranting further investigation.

## Author Contributions

GT, AI, MM, and SC drafted and wrote the manuscript. AI, CN, JP, M-VL, and SC performed the microbiological analysis. EC, NC, PK, and LP acquired the data for the study. All authors actively contributed to the data compilation and bibliography search, read, and proposed corrections to the manuscript.

## Conflict of Interest Statement

The authors declare that the research was conducted in the absence of any commercial or financial relationships that could be construed as a potential conflict of interest.
